# Spatial Distribution and Determinants of Early Resumption of Postpartum Sexual Intercourse among Postpartum Women in Ethiopia: A Multilevel Analysis

**DOI:** 10.1007/s00404-026-08380-9

**Published:** 2026-03-16

**Authors:** Mequanente Dagnaw, Meera Indracanti, Melaku Hunie Asratie

**Affiliations:** 1 Department of Epidemiology and Biostatistics, Institute of Public Health, College of Medicine and Health Sciences, University of Gondar, Gondar, Ethiopia; 2 Department of Medical Biotechnology, Institute of Biotechnology, University of Gondar, Gondar, Ethiopia; 3 Department of Medical Biotechnology, School of Allied and Healthcare Sciences, Malla Reddy University, Hyderabad, 500100 Telangana India; 4 Department of Women’s and Family Health, School of Midwifery, College of Medicine and Health Sciences, University of Gondar, Gondar, Ethiopia

**Keywords:** Resumption, Postpartum, Women, EDHS, Multilevel

## Abstract

**Introduction:**

Postpartum sexual health and practice need to be integrated into the current maternal healthcare services to address sexual health problems. However, postpartum sexual practice has received little attention and is not often discussed by healthcare providers during prenatal and postnatal care, even though evidence is scarce on the spatial distribution of early resumption of postpartum sexual intercourse in Ethiopia.

**Objective:**

The current study aimed to demonstrate the median time to resumption of postpartum sexual intercourse and the spatial distribution of early resumption of postpartum sexual intercourse in Ethiopia.

**Methods:**

A cross-sectional study was employed based on the Ethiopian demographic and health survey 2016 data, and 6456 postpartum period women were included. Arc GIS version 10.7 and SaTScan version 9.6 software were used. Mixed-effect analysis was done by STATA version 14 software. Bivariate analysis was done, and variables with a p value < 0.2 were taken as candidates for multilevel multivariable logistic regression. Intra Class Correlation Coefficient (ICC), Proportion Change in Variance (PCV), and Median Odds Ratio (MOR) were used for model comparison, and Adjusted Odds Ratio (AOR) with respect to a 95% confidence interval was used for declaring statistical significance. In the multivariable analysis, a p value ≤ 0.05 was considered as a cut point of statistical significance with the outcome variable.

**Results:**

The spatial distribution of early resumption of postpartum sexual intercourse was not random. Not married (Adjusted Odds Ratio (AOR = 0.31; 95% CI 0.25, 0.39), sex of child female (AOR = 0.87; 95% CI 0.81,0.93), protestant in religion (AOR = 0.68; 95% CI 0.58, 0.79), ever breastfeeding (AOR = 1.35; 95% CI 1.12, 1.63), and multiparity (AOR = 1.16; 95%, 1.03, 1.30) were variables significantly associated with early resumption of postpartum sexual intercourse.

**Conclusions:**

The spatial distribution of early resumption of postpartum sexual intercourse was not random. We need to give attention to those hotspot areas and factors significantly associated with early resumption of postpartum sexual intercourse. Reproductive and maternal health program managers and policymakers need to pay attention to those hotspot areas and significant variables to achieve the Sustainable Development Goal.

## Introduction

Having the first penetrative vaginal sexual intercourse after childbirth is characterized as resumption of postpartum sexual intercourse [1]. In the postpartum period, the resumption of sexual desire, sexual arousal, sexual intercourse, orgasm, and sexual satisfaction are attributes of postpartum sexual health [2]. The resumption of sexual intercourse after delivery is a significant feature of postpartum sexual health. The resumption of postpartum sexual intercourse at an inappropriate time dramatically affects the quality of life and self-esteem of postpartum women [3]. Gynecological complaints like sexual discomfort and dyspareunia due to incomplete healing of lacerations or episiotomy are mostly associated with early resumption of postpartum sexual intercourse [4, 5]. In addition, plenty of evidence showed that resumption of postpartum sexual intercourse in the early postpartum period is significantly associated with sexual morbidity, vaginal loosening, lack of sexual desire, genital tear, lack of vaginal lubrication, abnormal vaginal discharge, and difficulty in achieving orgasm [4, 6]. Consequently, resumption of postpartum sexual intercourse too soon predisposes to infection of reproductive organs due to vaginal abrasions and lesions [4]. Unfortunately, in addition to the aforementioned direct gynecological and obstetrical complications of early resumption of postpartum sexual intercourse, the bad consequences of unintended pregnancy and short interbirth interval are commonly displayed in developing countries [7].

Furthermore, early resumption of postpartum sexual intercourse without supplementation of effective contraceptive methods might cause unintended pregnancies that may result in numerous poor maternal and child health outcomes [8, 9]. Unintended pregnancy is a significant public health problem in Ethiopia, and evidence shows that the prevalence of unintended pregnancy in Ethiopia is 30% [10]. During the COVID-19 pandemic, the prevalence of unintended pregnancy was 47.17% [11], 19.5% [12], and 33.6% [13]. Notably, a research study conducted in developing nations showed that resumption of postpartum sexual intercourse in the early postpartum period is associated with a short interbirth interval, and this is dangerous for the health of the neonate and child health by increasing the occurrence of fever, measles, tetanus, and diarrhea [14]. Despite this, sexual activity is a principal topic in women’s health, and counseling on resumption of postpartum sexual intercourse in pregnant women has generally been overlooked by physicians and other healthcare staff [15]. Sexual practice during the postpartum period is an important element that has been identified in women’s healthcare, which needs increasing attention worldwide [6]. According to the World Health Organization (WHO) recommendations, as part of an overall assessment following childbirth, all women should be assessed regarding resumption of sexual intercourse and possible dyspareunia [16].

Though the World Health Organization (WHO) recommends that all women be evaluated regarding the resumption of sexual intercourse as a part of general assessment 2–6 weeks following delivery, little attention has been given by researchers, policymakers, and healthcare providers [16]. Besides, in most developing countries, many postpartum women do not get information or counseling about postpartum sexual health during the antenatal and postnatal period on when to resume sexual intercourse safely after delivery. Similarly, in Ethiopia, most studies conducted on women’s health during the postpartum period focused primarily on family planning utilization [17].

Having concrete data on the spatial distribution of early resumption of postpartum sexual intercourse is especially important to avert a substantial number of maternal, neonatal, and child mortality from different perspectives [18]. Therefore, the current study is based on the spatial distribution and determinants of early resumption of postpartum sexual intercourse in Ethiopia.

## Methods

### Study design, area, and period

We used secondary data from the Ethiopian Demographic and Health Survey (EDHS) conducted from January 18th of January 2016, to 27th of June 27th, 2016. A cross-sectional survey was conducted in all regions of Ethiopia (Tigray, Afar, Amhara, Oromia, Somali, Benishangul-Gumuz, Southern Nations, Nationalities and Peoples Region (SNNPR), Gambella, and Harari and two administrative councils—Addis Ababa and Dire Dawa. Currently, the total population of Ethiopia is 120 million, and 80% of this huge number lives in the rural parts of the nation.

### Data source and sampling procedure

For the current study, the data were accessed from the Ethiopian Demographic and Health Survey after being registered as an authorized user. The source population of the current study was all postpartum period women within 0–36 months (about 3 years) before the survey in Ethiopia, while all postpartum period women in the selected enumeration areas within 3 years before the survey were the study population. Using the 2007 Population and Housing Census as a sampling frame, a two-stage stratified cluster sampling procedure/technique was employed. In the first stage, 645 enumeration areas (EAs) were selected with probability proportional to the EA size and with independent selection in each sampling stratum. In the second stage, on average, 28 households were systematically selected. For the current study, a total weighted sample size of 6456 postpartum period women were included. The details of the sampling procedure can be accessed from the report of the measure DHS website (www.dhsprogram.com).

### Variables of the study

#### Dependent variable

***Early resumption of postpartum sexual intercourse (Yes/No):*** The variable duration of abstinence (m8) recoded as (Yes) early resumption of postpartum sexual intercourse when a postpartum period woman resumed sexual intercourse within 6 weeks of postpartum period and recoded as (No) when a postpartum period women not resumed sexual intercourse within 6 weeks of postpartum period [19].

***Independent variables:*** independent variables were categorized into two catalogs of socio-demographic-related factors (age of women, educational level, marital status, religion, media exposure, wealth status, community educational level, community media exposure level, and community poverty level), obstetrical and maternal health care related factors (parity, type of pregnancy, history of pregnancy termination, duration of breastfeeding, sex of child, knowledge on ovulation period, and place of delivery) (Table 1).

**Table 1:** Operational definitions of independent variables

Variables	Operational definition
Age	Age was a continuous variable at the beginning and now recoded as 15–24 = 1, 25–34 = 2 and 35–49 = 3 [20]
Marital status	Recoded never in union, living with partner, widowed, divorced, and no longer living together/separated = not currently married, and married has been kept as it is
Wealth status	The variable was created wealth index combined poorer and poor = poor, middle = middle, and richer and rich = rich (21)
Religion	Catholic and traditional followed were categorized into “others,” and the remaining were kept as they are
Media exposure	It was created by combining the variable frequency of reading newspapers, the frequency of listening radio, and the frequency of watching television
The community women's educational level	It was created form the variable v106 by the following steps first v106 recoded in to educated/yes and not educated/no, 2nd tab v001the recoded v106 [iw = wt], 3rd perform proportion by excel and save as csv coma delaminated, 4th open new STATA and import it and save as STATA education, 5th go to STATA data and combine the STATA by v001, 6th codebook education, 7th Recode < 50% as community women educational level “low” and ≥ 50% as community women educational level “High”
Community media exposure level	The step as above and recoded as < 50% = Low community media exposure level and ≥ 50% High community media exposure level
Community poverty level	The step was as above and recoded as < 50% = low community poverty level and ≥ 50% as high
Place of delivery	Generated by recoding other home, respondent home, and other as “home delivery” and else as government health facility delivery and private health facility

### Data management and analysis

#### Spatial analysis

ArcGIS version 10.7 and **SaTScan version 9.6** statistical software were used for exploring the spatial distribution/projection, global spatial autocorrelation, incremental autocorrelation, spatial interpolation/prediction, for identifying significant hotspot areas of early resumption of postpartum sexual intercourse.

#### Spatial autocorrelation analysis

The spatial autocorrelation (Global Moran’s I) is the correlation coefficient for the relationship between a variable and its surrounding values; it measures the overall spatial autocorrelation of early resumption of postpartum sexual intercourse [22]. It measures whether early resumption of postpartum sexual intercourse is clustered, dispersed, or randomly distributed in the study area (ref). Moran’s I is a spatial statistics measure used to measure spatial autocorrelation by taking the entire dataset and producing a single output value, which ranges from − 1 to + 1. The spatial autocorrelation coefficient is statistically significant when tested against the null hypothesis that the observed value differs from its expected value, which is 1/(n-1), where n is the number of points at the enumeration area level for which the autocorrelation is being computed. Moran’s I value ranges from -1 to 1 [23]. When Moran’s I values are close to − 1, it indicates the outcome is dispersed/shows a strong negative spatial autocorrelation, whereas I values close to + 1 indicate the outcome of interest is clustered/shows a strong positive spatial autocorrelation, and the outcome of interest is distributed randomly/there is no spatial autocorrelation if the I value is zero. A statistically significant Moran’s I (*p* < 0*.*05) leads to the rejection of the null hypothesis (early resumption of postpartum sexual intercourse is randomly distributed) and indicates the presence of spatial autocorrelation [24].

#### Hotspot analysis (Getis-Ord Gi.* statistics)

Getis-Ord Gi^*^ statistics were computed to measure how spatial autocorrelation varies over the study location by calculating the GI^*^ statistic for each area. The Z-score was computed to determine the statistical significance of clustering, and the *p value* was computed for the significance. Statistical output with high GI^*^ indicates “hot spot,” whereas low GI^*^ means a “cold spot.”

##### Spatial interpolation/spatial prediction

The technique was used to predict early resumption of postpartum sexual intercourse in the unsampled areas in Ethiopia based on sampled clusters/enumeration areas (EAs). There are different geostatistical and deterministic interpolation methods. Of those various methods/techniques, empirical Bayesian Kriging and ordinary Kriging are the best interpolation techniques, because they optimize the weight [24]. The ordinary Kriging spatial interpolation technique was used in the current study to predict early resumption of postpartum sexual intercourse in unobserved areas, because it had a small residual and mean square error.

##### Spatial scan statistical analysis

In the spatial scan statistical analysis, a Bernoulli-based model was used in which events at particular places were analyzed, whether there was early resumption of postpartum sexual intercourse or not, as 1/0. The scan statistics developed by Kulldorff and the SaTScan software version 9.6 were used to identify the presence of purely spatial early resumption of postpartum sexual intercourse clusters. Scan statistics were scanned gradually across the space to identify the number of observed and expected observations inside the window at each location. The scanning window with the maximum likelihood was the high-performing cluster, and a *p value* was assigned to this cluster. In this study, women whose early resumption of postpartum sexual intercourse had been the care were considered cases, and those who did not were considered controls to fit the Bernoulli model. The number of cases in each location had a Bernoulli distribution, and the model required data for cases, controls, and geographic coordinates.

#### Mixed-effect logistic regression analysis

In this study, both random and fixed-effect analysis was performed, and the analysis was done using STATA version 14 software. We applied the sampling weight to keep the representativeness of the study population to the overall enumeration area. Descriptive statistics, such as frequency and percent, were performed and presented in tables and graphs. Importantly, a multilevel multivariable logistic regression analysis was done by considering the hierarchical nature of the data*.* First, a bivariate multilevel logistic regression analysis was performed to find the crude odds ratio at a 95% confidence interval, and those variables statistically significant at 0.2 were used in the multilevel multivariable logistic regression analysis. Finally, multilevel multivariable logistic regression analysis was performed to estimate the adjusted odds ratio and random variation between clusters. Statistically significant variables at a *p value* less than 0.05 were reported with their 95% confidence interval.

In the multilevel multivariable logistic regression model, fixed-effect estimates measure the association between the odds of early resumption of postpartum sexual intercourse of individual- and community-level factors with a 95% confidence interval. Intra-cluster correlation coefficient (ICC) quantifies the degree of heterogeneity of early resumption of postpartum sexual intercourse between clusters. The proportion of change in variance (PCV) measures the proportion of the total observed individual variation that is attributable to the between-cluster variations [25]. The median odds ratio (MOR) measures the value between high- and low-risk clusters (EAs).

#### Ethical considerations

Since the study was a secondary data analysis of publicly available survey data from the MEASURE DHS program, ethical approval and participant consent were not necessary for this particular study. We requested the DHS program, and permission was granted to download and use the data for this study from http://www.dhsprogram.com. The Institutional Review Board approved procedures for DHS public-use datasets do not in any way allow respondents, households, or sample communities to be identified. There were no names of individuals or household addresses in the data file. The geographic identifiers only go down to the regional level (where regions are typically very large geographical areas encompassing several states/provinces). Each enumeration area (Primary Sampling Unit) has a PSU number in the data file, but the PSU numbers do not have any labels to indicate their names or locations. In surveys that collect GIS coordinates in the field, the coordinates are only for the enumeration area (EA) as a whole, and not for individual households, and the measured coordinates are randomly displaced within a large geographic area, so that specific enumeration areas cannot be identified.

## Results

### Socio-demographic factors of respondents in Ethiopia from the 2016 EDHS data

Among 6455.56 postpartum women, 3329.14 (51.57%) were within the age group of 25–34, 4013.1 (62.16%) had no formal education, 649.69 (93.71%) were currently married, 5004 (80.59%) were rural in residency, and 3016.00 (46.72%) were poor. Among all participants, 2694.39 (41.74%) were Muslim in religion, 4269.39 (66.14%) had no media exposure, 3392.64 (52.55%) had high community level women’s education, 3212 (51.73%) were in low community level media exposure, and 3158 (50.86%) were at low community level poverty (Table 2).

**Table 2:** Socio-demographic factors of participants in Ethiopia

Variables	Frequency	Percent
Age		
15–24	1,769.05	27.40
25–34	3,329.14	51.57
35–49	1,357.37	21.03
Educational status of women		
Have no formal education	4,013.1	62.16
Have a formal education	2,442.47	37.84
Marital status		
Currently not married	405.866	6.29
Currently married	6,049.69	93.71
Residency		
Urban	1205	19.41
Rural	5004	80.59
Wealth status		
Poor	3,016.00	46.72
Middle	1,313.51	20.35
Rich	2,126.06	32.93
Religion		
Others	217.40	3.37
Protestant	1340.14	20.76
Muslim	2694.39	41.74
Orthodox	2203.63	34.14
Media exposure		
No	4,269.39	66.14
Yes	2,186.17	33.86
Community women’s education level		
Low	3062.92	47.45
High	3392.64	52.55
Community media exposure level		
Low	3212	51.73
High	2997	48.27
Community poverty level		
High	3051	49.14
Low	3158	50.86

Others = Catholic, traditional followers

### Obstetrical and maternal health care services-related factors of participants in Ethiopia

Among 6456 postpartum period women, 5,298.22 (82.07%) were multiparity, 5883.49 (91.14%) were their pregnancy wanted, 5,912.25 (91.58%) had no history of pregnancy termination, and 4,426.72 (68.57%) were still breastfeeding. Among all participants, 3,250.91 (50.36%) were their child's sex male, 5,375.94 (83.28%) were knowledgeable on the ovulation period, and 4,342.41 (67.27%) were their place of delivery at home (Table 3).

**Table 3:** Obstetrical and maternal health care services related factors of participants

Variables	Frequency	Percent
Parity		
Primi parity	1,157.35	17.93
Multi parity	5,298.22	82.07
Type of pregnancy		
Wanted	5,883.49	91.14
Un wanted	572.08	8.86
History of pregnancy termination		
No	5,912.25	91.58
Yes	543.32	8.42
Duration of breastfeeding		
Not currently breastfeeding	1,751.19	27.13
Never breastfeed	277.65	4.30
Still breastfeeding	4,426.72	68.57
Sex of child		
Male	3,250.91	50.36
Female	3,204.65	49.64
Knowledge of the ovulation period		
Yes	5,375.94	83.28
No	1,079.62	16.72
Place of delivery		
Home	4,342.41	67.27
Government health facility	2,026.88	31.40
Private health facility	86.28	1.34

### Spatial distribution/projection of early resumption of postpartum sexual intercourse in Ethiopia

**Fig. 1:**
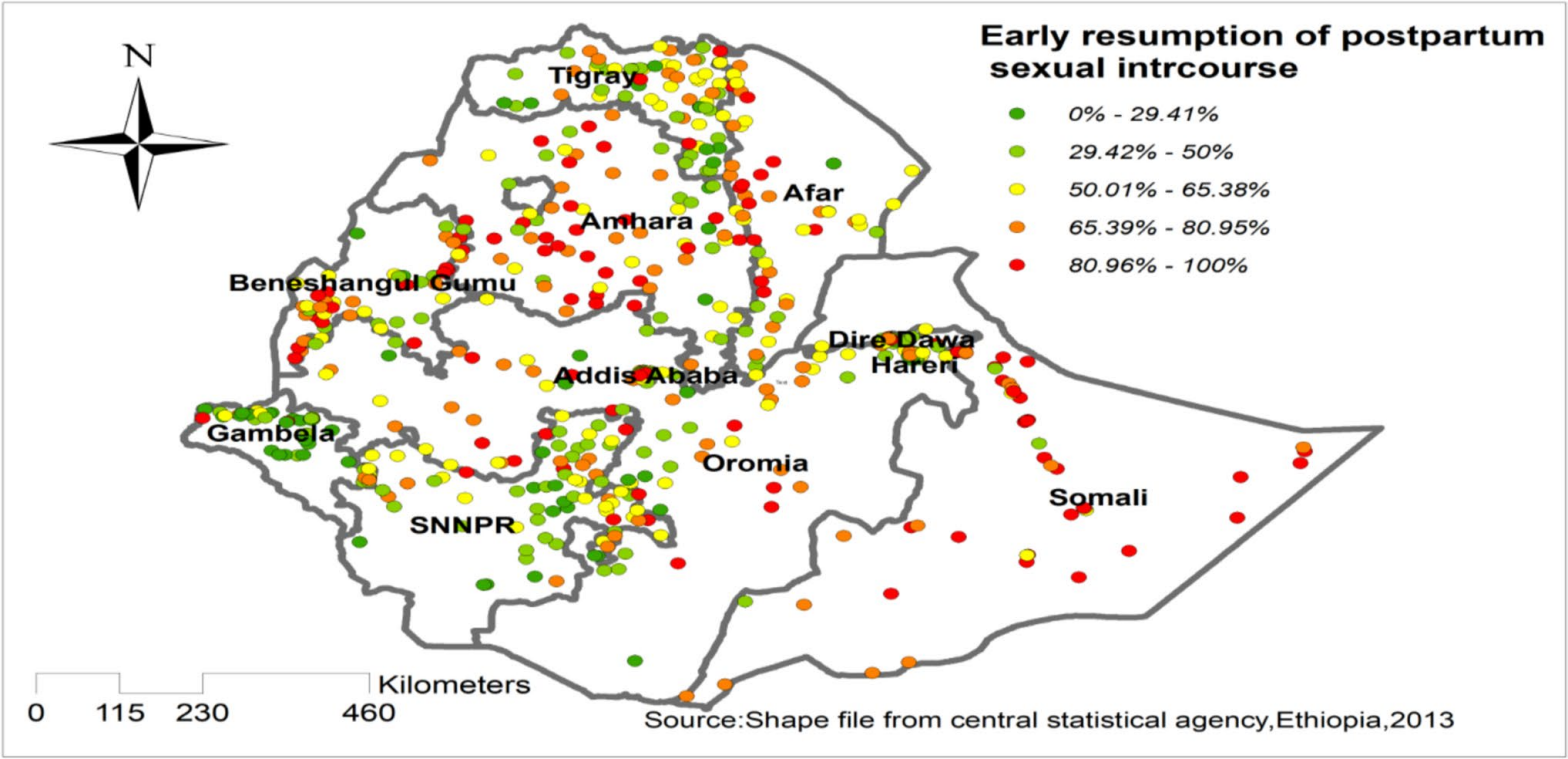
Spatial distribution/projection of early resumption of postpartum sexual intercourse in Ethiopia, EDHS 2016

The spatial projection of early resumption of postpartum sexual intercourse was done. On the map, each point represents one enumeration area with the proportion of early resumption of postpartum sexual intercourse in each cluster. The red color of the point on the map represents a high proportion of early resumption of postpartum sexual intercourse in the enumeration area, whereas the green color indicates an enumeration area with low proportion of early resumption of postpartum sexual intercourse. A higher proportion of early resumption of postpartum sexual intercourse was found in Amhara, Afar, and Somali. On the other hand, Tigray, SNNPR, and Gambela were characterized by a low proportion of early resumption of postpartum sexual intercourse **“**Fig. 1**”.**

### Spatial and incremental autocorrelation of newborn care in Ethiopia

**Fig. 2:**
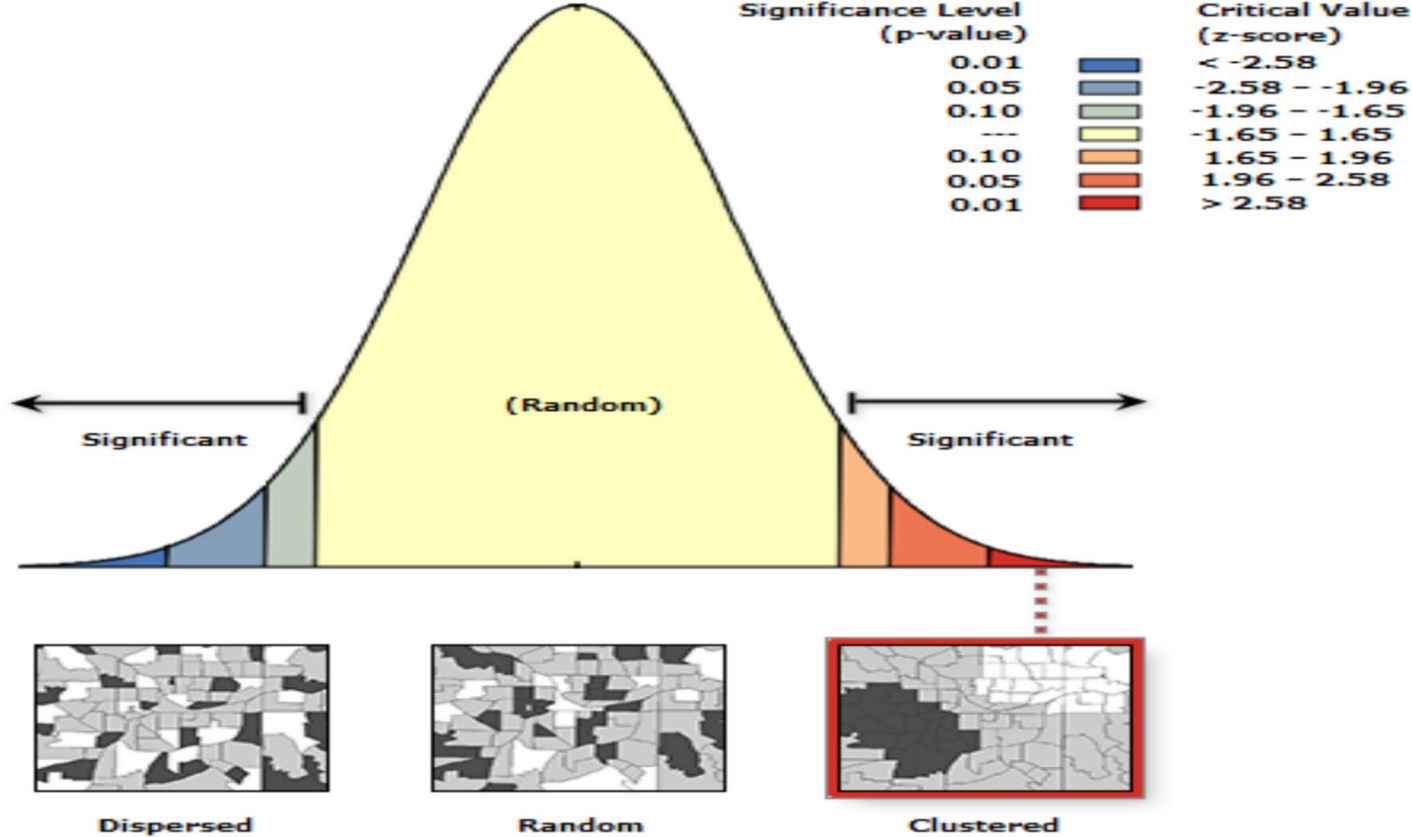
Spatial autocorrelation analysis of early resumption of postpartum sexual intercourse in Ethiopia, EDHS 2016

The clustered patterns (on the right sides) showed that a high rate of early resumption of postpartum sexual intercourse was observed. The *z* value showed that there is a clustered pattern with the probability of a chance < 1%. The bright red and blue colors to the end tails indicated that there is an increased significance level **“**Fig. 2**”.** The incremental spatial autocorrelation for a series of distances presented by a line graph with corresponding z-score was done to determine the average nearest neighbor and minimum and maximum distance band. A total of 10 distance bands were detected by a beginning distance of meters, 121,814, and the first maximum peak (clustering) was observed at 151,406.88 m.

### Hot spot analysis of early resumption of postpartum sexual intercourse

**Fig. 3:**
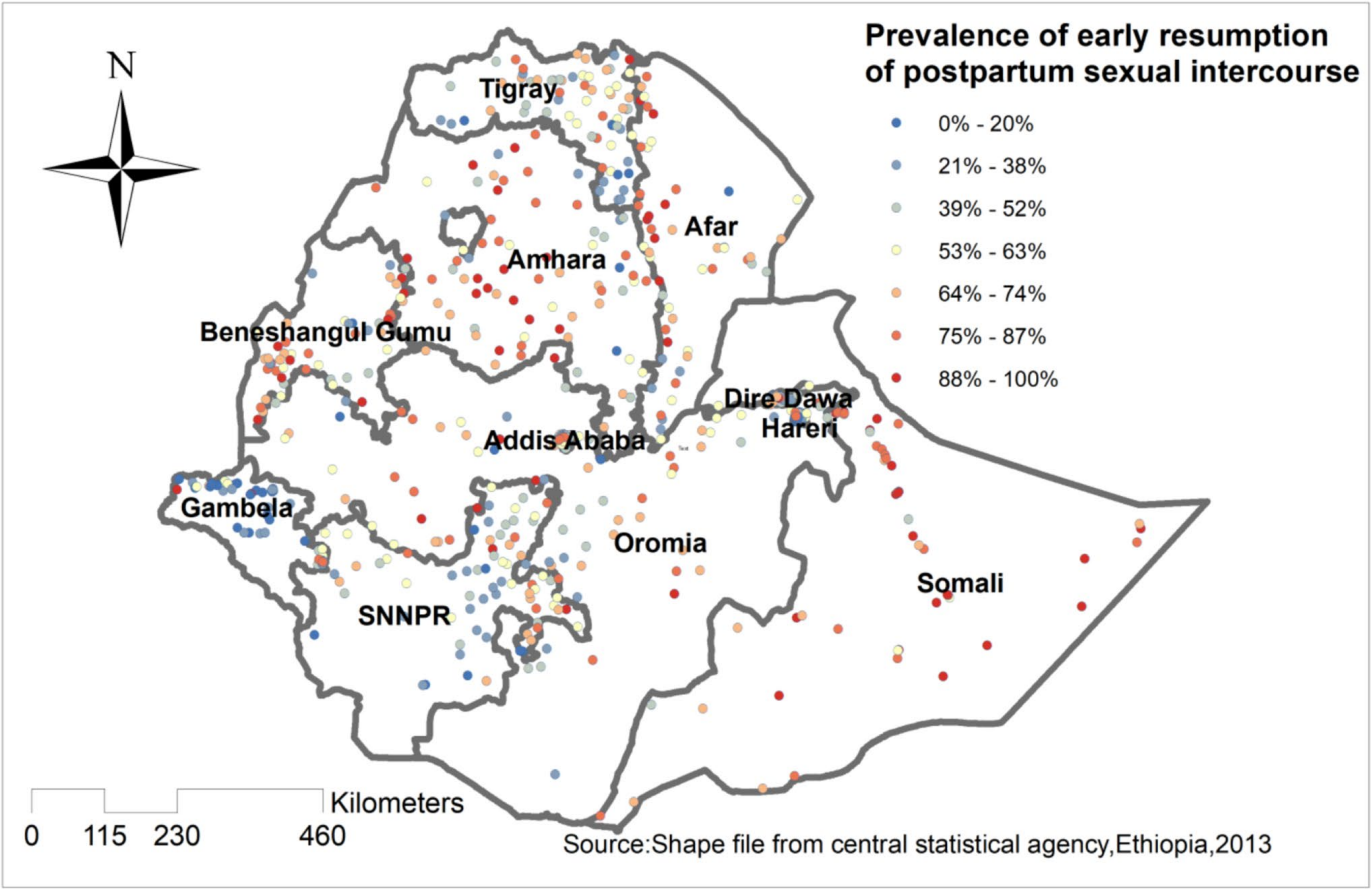
Hotspot analysis of early resumption of postpartum sexual intercourse in Ethiopia, EDHS 2016

Hotspot analysis was done to identify areas with a high probability for early resumption of postpartum sexual intercourse. The red color indicates areas with a high probability for early resumption of postpartum sexual intercourse, such as Amhara, Afar, Somali, and Addis Ababa. On the other hand, the blue color indicates early resumption of postpartum sexual intercourse is less likely performed “Fig. 3**”**.

### Spatial Interpolation/prediction of early resumption of postpartum sexual intercourse

**Fig. 4:**
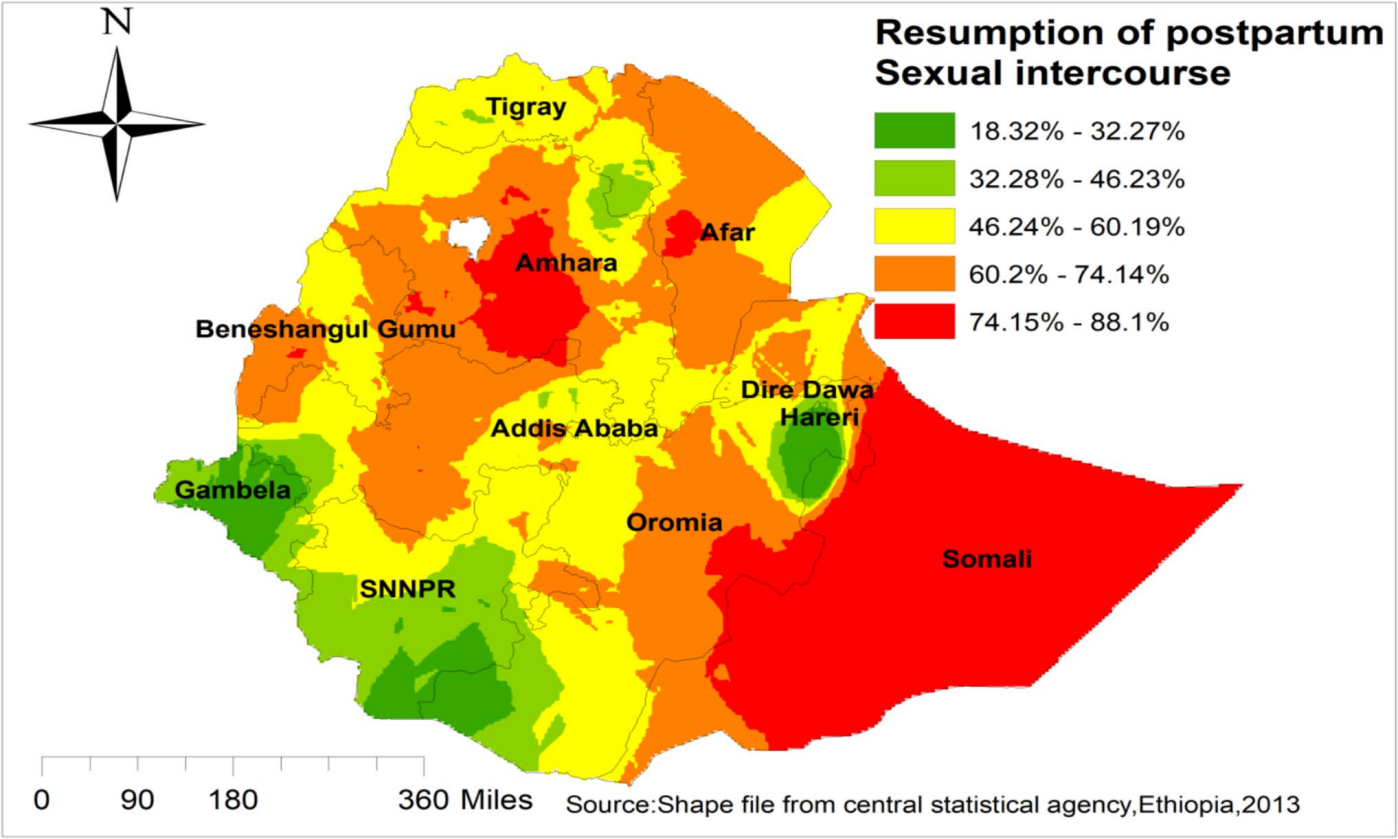
Spatial interpolation/prediction of early resumption of postpartum sexual intercourse in Ethiopia, EDHS 2016

The possibility of early resumption of postpartum sexual intercourse was increasing, while we moved from the green to the red-colored areas. The red and semi-red color predicts high possibility of early resumption of postpartum sexual intercourse, and the green color predicts low possibility areas of early resumption of postpartum sexual intercourse. Amhara, Somali, Afar, eastern Addis Ababa, and western Oromia were areas of highly predicted early resumption of postpartum sexual intercourse. On the other hand, Hareri, SNNPR, and Gambela were predicted fewer possibilities for early resumption of postpartum sexual intercourse “Fig. 4**”**.

### Spatial SaTScan Statistics Analysis of newborn care in Ethiopia

**Fig. 5:**
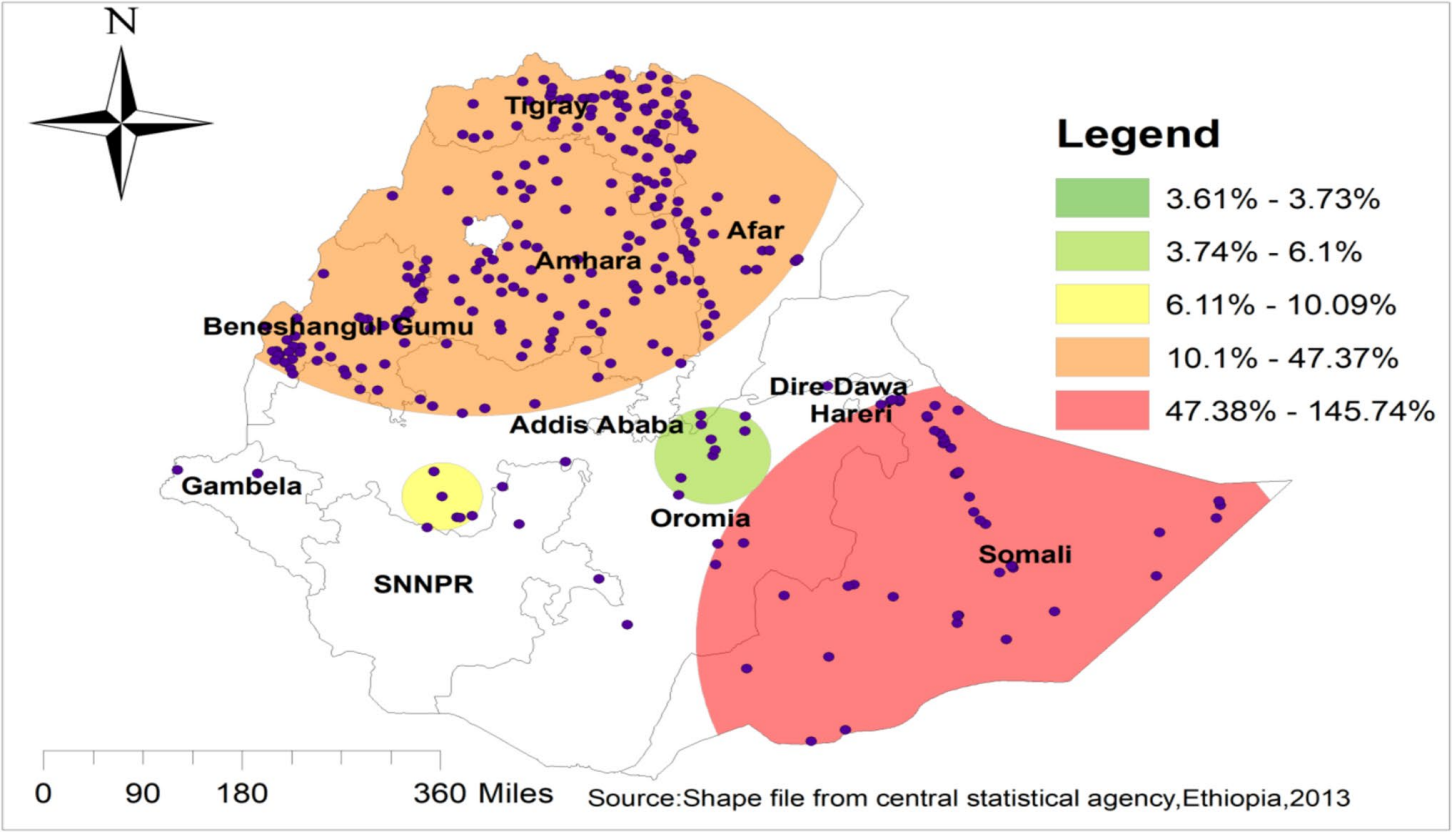
Significant clusters of early resumption of postpartum sexual intercourse spatial window in Ethiopia, EDHS 2016 plotted using ArcMap 10.7

There were primary and secondary clusters of early resumption of postpartum sexual intercourse in Ethiopia. Of the total clusters, 46 were significant primary clusters. These were in the entire Somali region centered at 5.589269 N, 44.175034 E) with a 451.86 km radius. Postpartum women who were found in the SaTScan window were 1.4 times more likely to resume postpartum sexual intercourse early (RR = 1.39, P value < 0.0001) **“**Table 4**, **Fig. 5**”.**

**Table 4:** Significant spatial clusters of newborn care in Ethiopia, EDHS 2016

Clusters	Enumeration areas (clusters) detected	Coordinate/radius	Population	Cases	RR	LLR	P value
1* [37]	138, 164, 85, 358, 146, 492, 92, 490, 543, 278, 171, 198, 95, 318,77, 187, 497, 556, 520, 629, 521, 588, 553, 458, 480, 208, 214, 251,573, 239, 269, 116, 22, 394, 378, 630, 568, 33, 277, 286, 527, 289,64, 439, 57, 186, 8, 210, 472	(5.589269 N, 44.175034 E) / 451.86 km	1079	882	1.39	145.738554	< 0.0001
2 [211]	253, 504, 612, 296, 258, 583, 268, 78, 98, 255, 528, 340, 188, 181, 638, 322, 584, 80, 425, 279, 312, 551, 640, 597, 400, 152, 156, 590, 636, 81, 327, 628, 84, 579, 52, 292, 479, 575, 45, 481, 355, 89, 461,163, 604, 199, 538, 430, 542, 424, 598, 226, 404, 129, 158, 512, 169,237, 94, 341, 550, 413, 132, 220, 605, 160, 392, 73, 623, 66, 384, 431, 99, 259, 298, 196, 117, 192, 136, 103, 143, 300, 516, 421, 602,415, 449, 127, 263, 456, 79, 382, 362, 361, 167, 134, 627, 235, 128,541, 511, 386, 442, 97, 351, 401, 585, 548, 403, 130, 172, 429, 38,515, 24, 591, 478, 200, 545, 455, 488, 615, 109, 498, 249, 120, 3,256, 533, 344, 332, 246, 559, 496, 176, 375, 544, 241, 206, 599, 410,36, 354, 189, 474, 616, 389, 150, 137, 35, 183, 364, 571, 460, 482,191, 611, 617, 348, 244, 494, 10, 18, 345, 531, 229, 184, 254, 267,350, 218, 368, 457, 510, 427, 55, 320, 569, 324, 65, 88, 285, 570,547, 209, 205, 409, 310, 572, 407, 124, 499, 178, 563, 335, 334, 621, 276, 595, 423, 620, 637, 581, 433, 70, 508, 203, 161, 317, 349, 6,294, 283, 517, 165, 399, 416, 17, 280, 440, 632, 295	(14.033877 N, 37.105923 E) / 545.49 km	3611	2348	1.18	47.374541	< 0.05

*significant primary clusters

### Multilevel logistic regression analysis of early resumption of postpartum sexual intercourse

Among the total 6,456 participants, 52.5% [95% *CI*: 51.3, 53.7] resumed postpartum sexual intercourse; the rest 47.4% did not resume postpartum sexual intercourse.

In the multilevel logistic regression model, variables with a *p* value of < 0.2 in the bi-variate analysis were considered for multivariable analysis. In the multivariable analyses, five variables such as marital status, sex of child, religion, duration of breastfeeding,, and parity, were statistically significant with the outcome variable early resumption of postpartum sexual intercourse.

The odds of early resumption of postpartum sexual intercourse was 69% less likely among women who were not married [Adjusted odds Ratio (AOR) = 0.31; 95% CI 0.25, 0.39] as compared to women who were married. The odds of early resumption of postpartum sexual intercourse were 13% less likely among women whose children were female (AOR = 0.87; 95% CI 0.81, 0.93) as compared to women with male children. Women with religion protestant and others were 32% and 35% less likely for early resumption of postpartum sexual intercourse (AOR = 0.68; 95% CI 0.58, 0.79) as compared to women with religion of orthodox. Women who ever breastfed had 1.35 times higher odds of early resumption of postpartum sexual intercourse (AOR = 1.35; 95% CI 1.12, 1.63) as compared to women who never breastfed. The odds of early resumption of postpartum sexual intercourse were 1.16 times higher among multipara women (AOR = 1.16; 95% CI 1.03, 1.30) as compared to primipara women “Table 5”.

**Table 5:** Multilevel logistic regression of early resumption of postpartum sexual intercourse in Ethiopia

Variables	Null model	Model I AOR [95% CI]	Model II AOR [95% CI]		Model III AOR [95% CI]
Age of women					
15–24		1			1.00
25–34		1.1[0.97, 1.14]			0.98[0.90, 1.08]
35–49		1.1[0.95, 1.16]			0.95[0.84, 1.07]
Sex of child					
Male		1			1
Female		0.85[0.80,0.92]^***^			0.87[0.81,0.93]^***^
Duration of breast feeding					
Never breast feed		1			1
Ever breast feed		1.18[0.99, 1.42]**			1.35[1.12, 1.63]***
0–6 month		0.80[0.66, 0.0.97]*			0.75[0.62, 1.92]
7–24 month		0.96[0.80, 1.15]**			1.11[0.93, 1.34]
25–35 month		0.93[0.75, 1.15]			1.07[ 0.86, 1.33]
Women educational status					
No formal education		1			1
Have formal education		0.84[0.78, 0.90] ***			1.01[0.92, 1.11]
Marital status					
Married		1			1
Not married		0.40[0.32, 0.49] ***			0.31[0.25, 0.39] ***
Media exposure					
No		1			
Yes		0.93[0.86, 1.00]			1.04[0.95, 1.14]
Religion					
Orthodox		1			1
Protestant		0.67[0.60,0.76]^***^			0.68[0.58,0.79]^***^
Muslim		1.14[1.05,1.23]^***^			1.09[0.98,1.23]
Other		0.61[0.46,0.82]^***^			0.65[0.47,0.91]^***^
Parity					
Primipara		1			1
Multipara		1.23[1.12, 1.35] ***			1.16[1.03,1.30]***
Knowledge on ovulatory period					
Yes		1			1
No		1.04[0.96, 1.14]			1.00[0.91,1.11]
Place of delivery					
Home		1			1
Government health facility		0.86[0.80,0.93]***			0.92[0.84,1.02]
Private health facility		0.73[0.59, 0.90]***			0.80[0.62, 1.03]
Residency					
Urban				1	1
Rural				1.09[0.99,1.19]	1.03[0.89,1.20]
Community-level women’s education					
Low				1	1
High				1.23[0.78,1.5]	1.43[0.79,1.67]
Community-level media exposure					
Low				1	1
High				2.67[1.88,2.97]	1.65[0.56,0.87]
Community-level poverty					
Low				1	1
High				0.86[0.56,1.45]	1.24[0.83,1.46]
Random effect					
Community-level variance	68.9%	68.5%		63.7%	65.0%
ICC	17.3%	17.2%		16.2%	16.5%
MOR	2.14[1.91,2.39]	2.14		2.06	2.08
PCV	Reference	58%		7.5%	5.6%
Model fit statistics					
Log likely Hood ratio	−4177.7573	−3995.8486		−4166.9733	−3989.1801
Deviance (−2 Log likelihood ratio)	8355.5146	7991.6972		8333.9466	7978.3602

*AOR *adjusted odds ratio, *ICC * intraclass correlation coefficient, *CI * confidence interval, *MOR * median odds ratio, *PCV * proportion change in variance

** = 0.001 < *p* < 0.005, and *** = *P* < 0.001

## Discussion

Early resumption of postpartum sexual intercourse has an adverse outcome on the health of women, and indirectly, unintended pregnancy might happen, affecting both the health of women and the delivered baby [26]. The spatial distribution of early resumption of postpartum sexual intercourse varied across different regions of Ethiopia. Significant hotspot areas of early resumption of postpartum sexual intercourse are identified in Amhara, Afar, Somali, and Addis Ababa. From the spatial interpolation/prediction, Amhara, Somali, Afar, eastern Addis Ababa, and western Oromia are areas of highly predicted early resumption of postpartum sexual intercourse. All those variations in the spatial distribution of early resumption of postpartum sexual intercourse might be due to the discrepancy in maternal healthcare services like antenatal care, health facility delivery, and postnatal care services [27, 28]. The other possible reason for this discrepancy in the spatial distribution of early resumption of postpartum sexual intercourse is due to the variation in socio-cultural factors across regions of Ethiopia [29].

Regarding the predictors of early resumption of postpartum sexual intercourse, a total of five variables, marital status, sex of child, religion, duration of breastfeeding, and parity are statistically significant in the multivariable analysis.

Not-married women are 69% less likely early resume in postpartum sexual intercourse as compared to women who are married. The possible explanation might be due to women who are not married having less probability of sexual contact with male partners/husbands, as they have no partner/husbands. Women with a female child are 13% less likely to resume early postpartum sexual intercourse as compared to women with male children. The possible explanation could be that religiously, women with a female child should refrain from postpartum sexual intercourse till the baptism of their baby, which is 80 days after delivery, whereas women with a male child have a limit for baptism is 40 days; therefore, this condition makes women with a female child less likely to resume postpartum sexual intercourse in the early period [26].

Women with the Protestant religion and others are 32% and 35% less likely of early resumption of postpartum sexual intercourse, respectively, as compared to women with the religion of orthodox. The possible explanation could be due to the difference in religiously induced education among Protestant followers. Women who have ever breastfed are 1.35 times more likely of early resuming postpartum sexual intercourse compared to women never breastfed. Multipara women are 1.16 times more likely to have early resumption of postpartum sexual intercourse as compared to primipara women. The possible explanation could be due to multipara women having a desire for more children, and they might initiate postpartum sexual intercourse early.

## Limitations and strengths of the study

The main strength of this study was the use of the weighted nationally representative data with a large sample, which makes it representative at national and regional levels. Therefore, it can be generalized to all postpartum period women during the study period in Ethiopia. Moreover, the use of a shared frailty model that considered the nested nature of the EDHS data and the variability within the community to get a reliable estimate and standard errors (SEs). However, it is not free of limitations that have resulted from the use of secondary data. Some important confounders, such as the health service quality and behavioral factors, are missed.

## Conclusions

The study showed that the spatial distribution of early resumption of postpartum sexual intercourse varied across regions of Ethiopia. The hotspot areas of early resumption of postpartum sexual intercourse were located in Amhara, Afar, Somali, and Addis Ababa. Besides, not being married, sex of child female, religion protestant, and other factors were negatively associated with early resumption of postpartum sexual intercourse. On the other hand, ever breastfeeding and multiparity were positively associated with the outcome variable. Therefore, policymakers and governmental and non-governmental organizations could strengthen the effort towards reproductive health services and should design effective public health interventions in the identified hotspot areas to reduce the incidence of early resumption of postpartum sexual intercourse and its related morbidity and mortality.

## Data Availability

All output-based data are available within the manuscript, and additionally, the dataset can be accessed from.

## Abbreviations

*AOR*: Adjusted odds ratio
*CI*: Confidence interval
*DHS*: Demographic and health survey
*MOR*: Median odds ratio
*LLR*: Log likelihood ratio
*NDSs*: Newborn danger signs
*RR*: Relative risk
*ICC*: Intra-class correlation coefficient
*PCV*: Proportion change in variance
